# Strangulation of intraperitoneal kidney transplant by fallopian tube

**DOI:** 10.1093/jscr/rjab347

**Published:** 2021-10-25

**Authors:** Atta Nawabi, Perwaiz Nawabi, Babak Mohammadian, Jill Jones

**Affiliations:** Department of Surgery, The University of Kansas, Kansas City, KS, USA; Department of Surgery, The University of Kansas, Kansas City, KS, USA; Kansas City University, Kansas City, MO, USA; University of Kansas Health System, Kansas City, KS, USA

## Abstract

Renal allograft strangulation is a rare complication following simultaneous kidney pancreas transplant, often causing graft loss. This case report represents the first documented case of a 35-year-old female who developed renal graft strangulation around the left fallopian tube. Our case outlines a new complication that contributes to graft loss concerning iliac fossa anatomy and variations in female patients, as well as surgical considerations that need to be made prior to transplantation. We recommend measurement of the grafted renal vessels within the iliac fossa and respective surroundings structures to allow for the ideal positioning of the grafted organ.

## INTRODUCTION

Simultaneous pancreas kidney (SPK) transplantation remains the gold standard and definitive treatment in insulin-dependent diabetic patients with end-stage renal disease. Patient selection for SPK is determined by many factors, including age, GFR, insulin requirement, BMI and comprehensive multidisciplinary. Commonly, the donor kidney and pancreas are transplanted intraperitoneally through a vertical lower midline incision. Intraperitoneal placement takes advantage of the vast intraperitoneal lymphatic circulation to allow for greater permeability and absorption of peripancreatic secretions. In most cases, the kidney is placed in the left iliac fossa and the pancreas in the right iliac fossa.

Routine follow-up and monitoring are standard for every patient undergoing SPK transplantation. Postoperative tapering of immunosuppressive therapy is done during this time to prevent organ rejection while mitigating the adverse effects of medication. Unfortunately, urinary tract infections (UTIs) are among the most common bacterial infections occurring in transplant recipients [1]. We have previously reported the first case of a 29-year-old female with sudden development of late-onset renal vein thrombosis due to compression by an ovarian cyst [2]. Here, we present a 35-year-old female who underwent SPK transplantation complicated by strangulation of the renal vein and artery of the transplanted kidney by the fallopian tube. This is the first documented case of its kind.

## THE CASE

**Figure 1:**
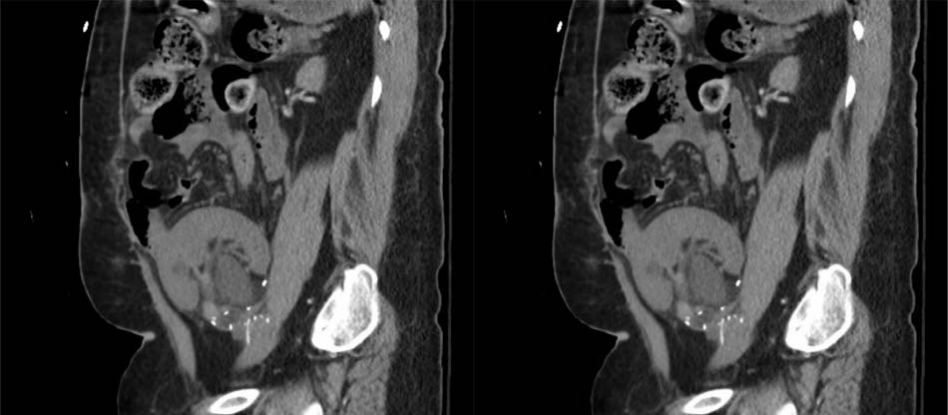
Coronal view of the CT abdomen pelvis without contrast showing hydronephrosis of the donor’s kidney.

**Figure 2:**
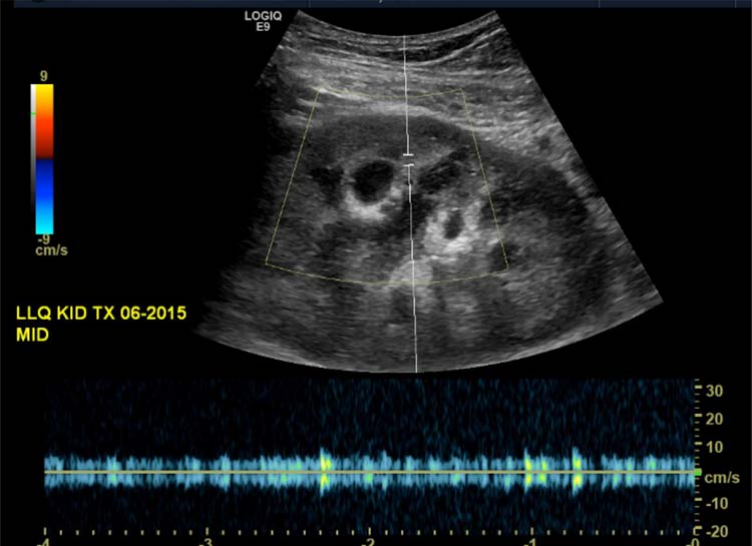
Ultrasound of the transplanted kidney showing no blood flow within the main renal artery or vein.

**Figure 3:**
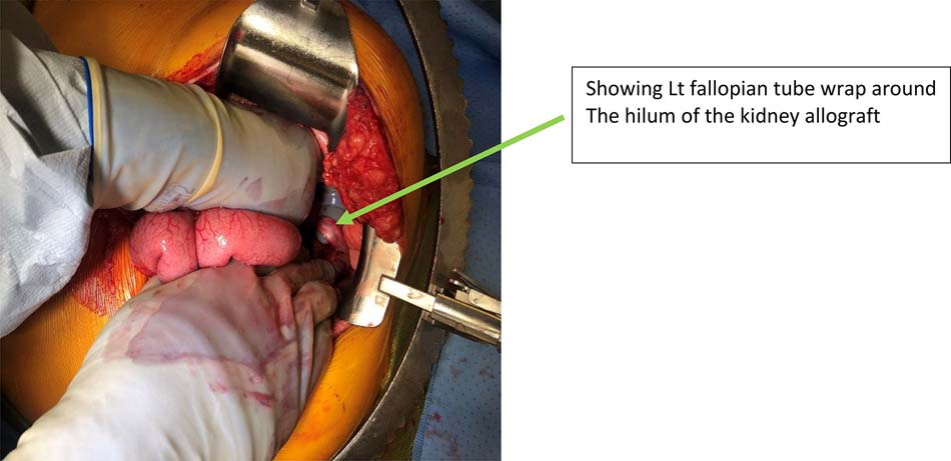
Intraoperative finding of Lt Fallopian tube strangulated the kidney allograft.

**Figure 4:**
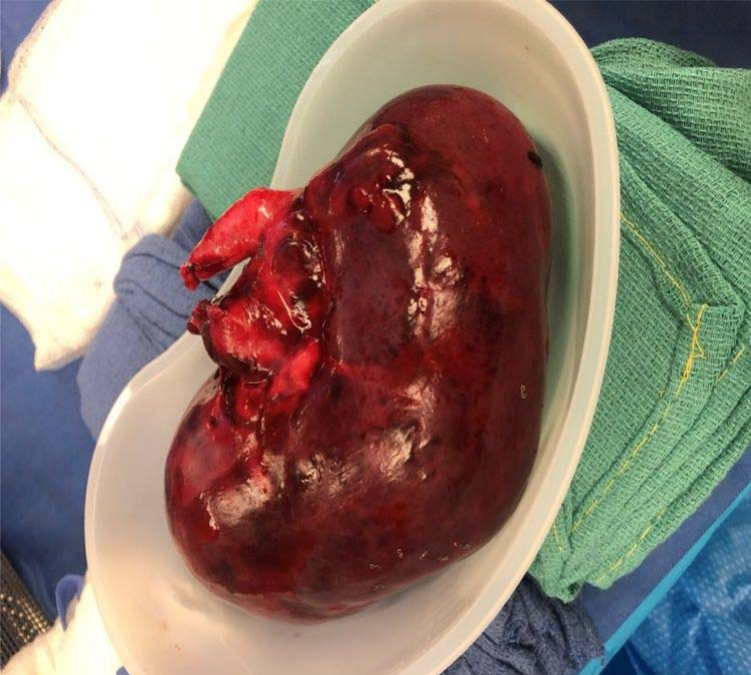
Ischemic and necrotic kidney allograft.

Our patient is a 35-year-old female with a history of Type 1 diabetes and hypertension who underwent SPK transplantation in 2015. The pancreas transplant was performed with systemic venous drainage and enteric exocrine drainage. Kidney transplantation was performed intraperitoneally in the left lower quadrant, with standard anastomoses performed between left external iliac vessels and renal artery/vein [1]. She presented to the emergency room 5 years after her transplantation with 24 h of severe Lt lower abdominal pain, nausea and vomiting with tachycardia and leukocytosis up to 22.6. The patient was diagnosed with sepsis secondary to UTI. Upon evaluation, she was anuric, and her creatinine was 5.45 mg/dl from 2.15. Computed tomography (CT) abdomen pelvis without contrast showed hydronephrosis of the donor kidney (Fig. 1). Doppler ultrasound was performed and showed no flow to the donor kidney (Fig. 2). She was urgently taken to the operating room for exploration. Intraoperatively, we found that the renal artery and vein of the transplanted kidney was strangulated by the left fallopian tube and respective ovary (Fig. 3). The transplanted kidney was noted to be gangrenous and ischemic (Fig. 4). In addition, intraoperative Doppler confirmed no arterial blood flow, and the decision was made to perform a transplant nephrectomy. The remainder of her hospital course was unremarkable. The patient was subsequently re-listed for a kidney transplant.

## DISCUSSION

Strangulation of a transplanted kidney by the fallopian tube is a rare and devastating complication. Early detection and intervention are vital as graft loss occurs rapidly. Our case outlines a new complication that contributes to graft loss concerning iliac fossa anatomy and variations in female patients. Anatomically, the ovary is suspended by the infundibulopelvic ligament and the fallopian tubes are attached to the broad ligaments [3, 4]. Both the fallopian tubes and ovaries are considered part of the uterine adnexa and lie within the contents of the iliac fossa [5]. Previously, we documented a case of an ovarian cyst compressing the renal vein of a grafted kidney in a young woman, ultimately causing renal vein thrombosis [2], and others documented intestinal obstruction caused by the fallopian tube [5, 6]. Such rare but recurrent complications shed light on surgical approaches and female-specific considerations that need to be made intraoperatively. We propose that optimal measurement of the grafted renal vessels within the iliac fossa would allow proper positioning of the grafted organ. Moreover, special attention should be given to individuals with previous surgical history, especially previously abdominal transplantation. Finally, we propose that the intraperitoneal transplantation of the pancreas should be performed in the right lower quadrant with retroperitoneal transplantation of the kidney.

## CONFLICT OF INTEREST STATEMENT

None declared.
